# Disrupting neural activity related to awake-state sharp wave-ripple complexes prevents hippocampal learning

**DOI:** 10.3389/fnbeh.2012.00084

**Published:** 2012-12-04

**Authors:** Miriam S. Nokia, Jarno E. Mikkonen, Markku Penttonen, Jan Wikgren

**Affiliations:** Department of Psychology, University of JyväskyläJyväskylä, Finland

**Keywords:** classical conditioning, ripple, theta, learning, hippocampus

## Abstract

Oscillations in hippocampal local-field potentials (LFPs) reflect the crucial involvement of the hippocampus in memory trace formation: theta (4–8 Hz) oscillations and ripples (~200 Hz) occurring during sharp waves are thought to mediate encoding and consolidation, respectively. During sharp wave-ripple complexes (SPW-Rs), hippocampal cell firing closely follows the pattern that took place during the initial experience, most likely reflecting replay of that event. Disrupting hippocampal ripples using electrical stimulation either during training in awake animals or during sleep after training retards spatial learning. Here, adult rabbits were trained in trace eyeblink conditioning, a hippocampus-dependent associative learning task. A bright light was presented to the animals during the inter-trial interval (ITI), when awake, either during SPW-Rs or irrespective of their neural state. Learning was particularly poor when the light was presented following SPW-Rs. While the light did not disrupt the ripple itself, it elicited a theta-band oscillation, a state that does not usually coincide with SPW-Rs. Thus, it seems that consolidation depends on neuronal activity within and beyond the hippocampus taking place immediately after, but by no means limited to, hippocampal SPW-Rs.

## Introduction

In the mammalian brain, the hippocampus is involved in various forms of learning and memory and is especially responsible for declarative (episodic) memory (Scoville and Milner, 1957; Squire, 2004). After new information has been encoded during the initial learning event, it then needs to be consolidated into long-term memory (Buzsaki, 1989). Consolidation is thought to happen mostly during sleep periods immediately following the training event (Diekelmann and Born, 2010; O'Neill et al., 2010), but also during “idle” moments interspersed with active training while awake (Jadhav et al., 2012). It is suggested that during memory consolidation recently acquired information is transferred from the hippocampus to scattered neocortical networks via synchronized oscillatory activity (Sirota et al., 2003; Montgomery et al., 2008).

The electrophysiological activity of the hippocampus is characterized by two distinct oscillations produced by synchronous firing of thousands of cells: rhythmic slow activity called theta (~4–8 Hz, Buzsáki, 2002) and high-frequency (~200 Hz), high-amplitude bursts of cell firing coupled with slower-frequency sharp waves (sharp wave-ripple complex, SPW-R), (Buzsaki, 1986; Chrobak and Buzsaki, 1996). Currently, the most influential view of the role of hippocampal oscillations in learning (Buzsaki, 1989) is that theta activity reflects a state in which the animal engages in behaviors directed at a specific target in the surroundings (i.e., selective attention). Thus, theta represents the “read” or encoding state of the learning process. In contrast, SPW-Rs occur in a state where the animal is not paying specific attention to its surroundings. During SPW-Rs, (place) cell firing in the hippocampus closely resembles that recorded during previous events (Foster and Wilson, 2006; Diba and Buzsaki, 2007; Davidson et al., 2009; Karlsson and Frank, 2009; Gupta et al., 2010), presumably reflecting replay and consolidation of recently acquired spatial information. Indeed, disrupting SPW-Rs following training on a spatial memory task hinders consolidation (Girardeau et al., 2009; Ego-Stengel and Wilson, 2010; Jadhav et al., 2012). However, presenting training stimuli contingent upon ripples accelerates learning, suggesting that the hippocampus is at its most responsive state immediately after ripples ensuring enhanced encoding of the stimuli (Nokia et al., 2010).

Whether hippocampal SPW-Rs and the replay of events *per se*, and/or the information flow between the hippocampus and cortical structures associated with them, is crucial for consolidation remains uncertain. To address this issue, we trained adult New Zealand White rabbits in trace eyeblink conditioning, a hippocampus-dependent associative learning task (Kim et al., 1995; Takehara et al., 2003). A light was presented to the rabbits during the inter-trial interval (ITI), when awake, either during SPW-Rs to disrupt neural activity normally related to it or irrespective of the ongoing neural state. We expected that disrupting SPW-R -related brain activity would disrupt learning.

## Materials and methods

### Subjects

The subjects were 33 adult New Zealand White rabbits (Lidköping's Kaninfarm, Lidköping, Sweden) aged ~4 months and weighing ~3 kg at the time of surgery. The rabbits were housed in individual metal cages at the animal research unit of the University of Jyväskylä. Food and water were freely available, and room temperature and humidity were controlled. The rabbits were maintained on a 12/12 h light/dark cycle, with lights on at 6.00 a.m. All procedures were conducted during the light portion of the cycle. All procedures were implemented in accordance with the European Communities Council Directive (86/609/EEC) on the care and use of animals for research purposes.

### Surgery

Subcutaneous injections of an analgesic solution [0.1 ml of 0.3 mg/ml buprenorphine (Temgesic, Schering-Plough Europe, Brussels, Belgium) diluted in 0.9 ml of 0.9% NaCl, dose: 2 ml] and of an anti-inflammatory drug [50 mg/ml carprofen (Rimadyl vet, Pfizer Inc. Animal Health, Espoo, Finland), dose: 0.1 ml/kg] was given 30 min preceding the onset of the surgery. The rabbits were anesthetized with an i.m. injection of ketamine-xylazine cocktail [7.8 ml of 50 mg/ml Ketaminol vet (Intervet International B.V., Boxmeer, Netherland) mixed with 2.8 ml of 20 mg/ml Narcoxyl vet (Intervet International B.V.)]. A dose of 0.8 ml/kg of the cocktail was injected i.m. before surgery. During surgery, additional 0.8 ml-doses of either the cocktail or ketamine alone were injected subcutaneously approximately every 20 min. At the beginning of the surgery, the rabbit was placed in a stereotaxic instrument (Kopf Instruments, Tujunga, CA, USA) with the bregma 1.5 mm higher than lambda. Eyedrops were then administered to prevent the animal's eyes from drying. A longitudinal incision was made to the scalp and four stainless-steel anchoring screws (5 mm anterior and 5 mm lateral to the bregma; 10 mm posterior and 5 mm lateral to the bregma) were attached to the skull. The screws were connected together and they served as a reference for the electrophysiological recordings.

Three monopolar recording electrodes made of Teflon-insulated stainless steel wire (bare diameter 125 μm, tip length ~200 μm) mounted inside a 27-gauge hypodermic stainless steel tubing were chronically implanted into the right hippocampus 5 mm posterior and 4/5/6 mm lateral to the bregma. During implantation, local-field potentials (LFPs) were monitored to define the preferred depth of the electrode (bregma −6.5 to 7.2 mm). Finally, the electrodes were attached to a pin connector and the whole construction cemented in place with dental acrylic.

Metoclopramide [dose 0.1 ml/kg, concentration 5 mg/ml; Primperan (Sanofi Winthrop Industrie, Quétigny, France)] was injected subcutaneously immediately after the surgery to facilitate normal feeding and drinking. Analgesic (buprenorphine, see above for details) was administered every 8 h for the next 24–48 h depending on the recovery rate of the animal. At least 1 week was allowed for post-surgical recovery.

### Conditioning procedure

**Figure 1:**
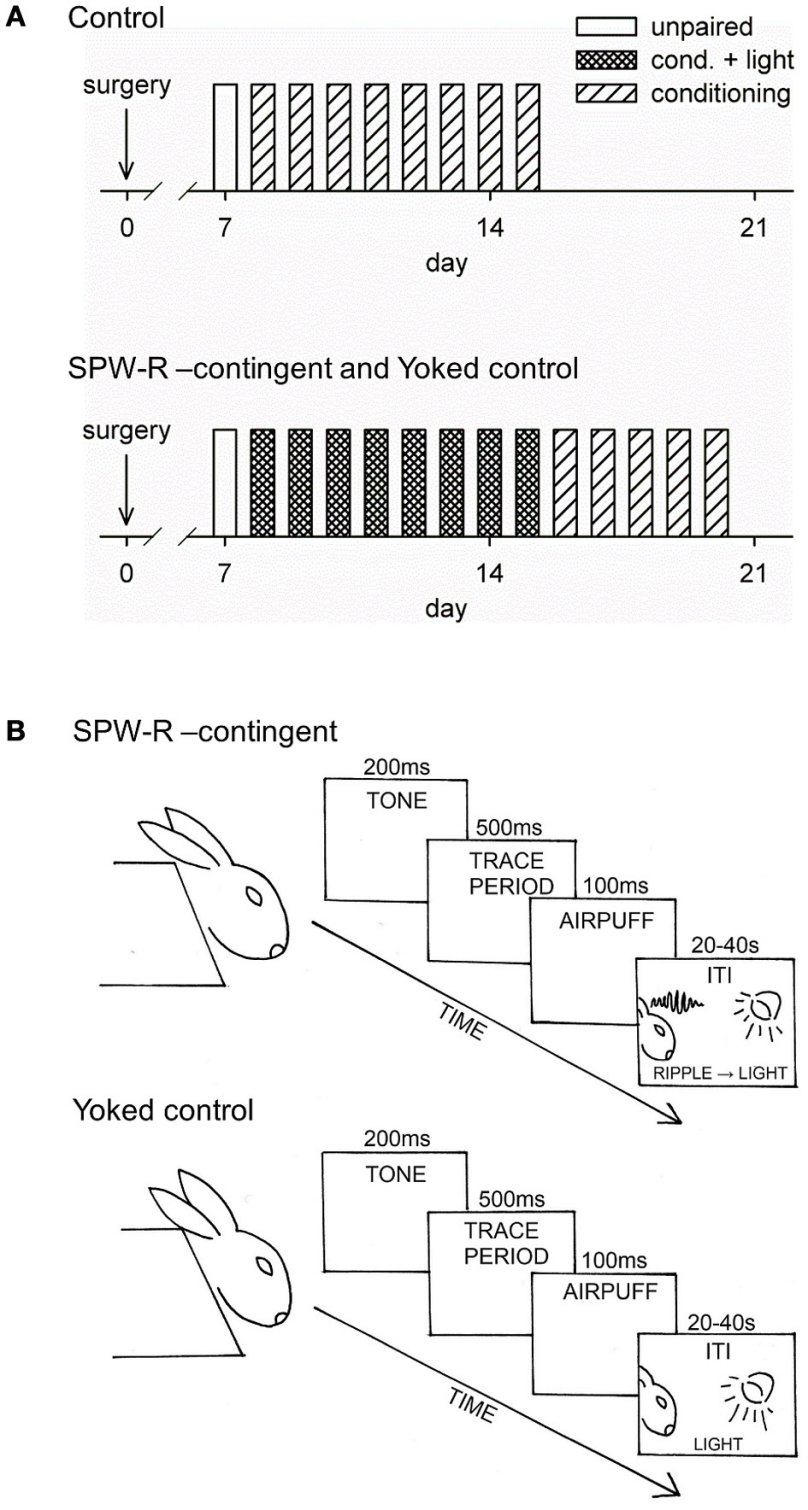
**Conditioning procedure.** Training over days for the control group (top) and for the SPW-R-contingent (R) and yoked control (Y) (bottom) groups is depicted in **(A)**. All animals initially received one unpaired session followed by 8 daily sessions of trace eyeblink conditioning. The conditioning sessions consisted of 60 trials with an average inter-trial interval (ITI) of 30 s. During a single trial, the tone-CS onset preceded the airpuff-US onset by 700 ms, thus creating a 500-ms stimulus-free trace period. Animals from the Y and R groups were grouped into pairs and trained together (see **B**). During the ITI, a flash of light was presented every time a ripple was detected in the animal assigned to the R group. After this, animals in the R and Y groups were trained for another 5 daily sessions now omitting the light.

The conditioning procedure is depicted in Figure 1. The rabbits were placed in a Plexiglas restraining box allowing free movement of the head (Schneiderman et al., 1962) and located in a ventilated, electrically insulated, and sound-attenuated conditioning chamber for ~20 min to familiarize them with the experimental situation. To examine responses to the flash of light before any conditioning, a separate test session was conducted with a subgroup of animals, where the light was presented contingent upon SPW-Rs or irrespective of the oscillatory state. Hippocampal responses were similar whether the light was presented contingent upon SPW-Rs or irrespective of the oscillatory state (data not shown). Experimental sessions were conducted once per day on consecutive days.

The stimuli used in trace eyeblink conditioning were a 4 kHz, 85-dB, 200-ms tone (conditioned stimulus, CS) and a 100-ms corneal airpuff (0.35 bar source pressure, SPL 64 dB) delivered through a nozzle (inner diameter 2 mm) placed approximately 1 cm away from the eye (unconditioned stimulus, US). A fan located inside the conditioning chamber behind the rabbit created a steady background noise of approximately 65 dB. E-Prime software (Psychology Software Tools Inc., Pittsburgh, PA, USA) was used to control the presentation of the tone-CS and the airpuff-US.

Rabbits with successful electrode placement in the hippocampus (i.e., showing SPW-Rs) were randomly assigned to Yoked control (Y, *n* = 12) and SPW-R-contingent (R, *n* = 12) groups. The rest were assigned to the Normal control (N, *n* = 9) group. All animals initially received eight daily sessions of trace eyeblink conditioning. Animals from the Y and R groups were grouped into pairs and trained together. The conditioning sessions consisted of 60 trials with an average ITI of 30 s (range 20–40 s). During a single trial, the tone-CS onset preceded the airpuff-US onset by 700 ms, thus creating a 500-ms stimulus-free trace period. During the ITIs, a flash of light from three light-emitting diodes (LEDs) placed in front of the animal's head was presented every time a SPW-R was detected in the animal assigned to the R group. Thus, simultaneously trained animals in the Yoked control group received the same light stimulation irrespective of their neural state. Animals in the N group received conditioning as described above but no presentations of light. In the next phase, animals from the R and Y groups were trained for another five daily sessions, but this time without the presentation of light stimuli.

### Recordings and data-analysis

Eyeblinks were recorded using an infrared detector (Ryan et al., 2006). To acquire neural measures, a low-noise pre-amplifier was directly attached to the electrode coupler anchored with dental acrylic to the rabbit's head. A flexible, insulated cable was used to connect the animal to the amplifier (Axon Cyberamp 380, Molecular Devices Corporation, Union City, CA, USA). All data were recorded with AxoScope (Molecular Devices Corporation) software and digitized (Digidata 1322A, Molecular Devices Corporation) using a 2 kHz sampling rate. Before digitization, the LFPs were band-pass-filtered between 1 and 400 Hz.

Clampfit (Molecular Devices Corporation), MATLAB (The MathWorks Inc., Natick, MA, USA), and SPSS (SPSS Inc., Chicago, IL, USA) were used for data analysis.

#### Conditioned responses

The occurrence of eyeblinks was scored using a custom-made protocol in MATLAB by three-independent reviewers unaware of the origin of the signal they were scoring. Trials with eyeblinks during a 100-ms period immediately preceding the CS-onset were rejected. Eyeblinks during the last 250 ms of the trace period were counted as conditioned responses (CRs). Eyeblinks elicited by the light presented to the SPW-R-contingent and yoked control groups during the ITI were analyzed in the same manner as CRs.

Not all animals are able to learn trace eyeblink conditioning, and those that do learn, according to our prior experience, usually do so within the first 8 daily sessions. To exclude the possibility that any learning impairments possibly evident during the first phase of the experiment would be attributable to a general inability to learn, subjects that made less than ~30% CRs within at least one session by the end of all training were excluded from further analysis (2/12 in the R group, 3/12 in the Y, and 3/9 in the N group).

#### Online SPW-R-contingent triggering

**Figure A1:**
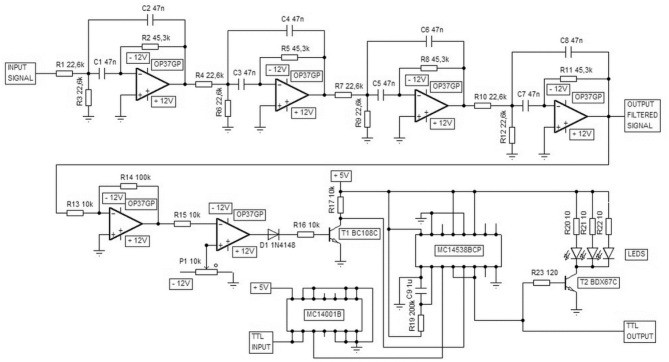
**A custom-made device was used for ripple-contingent triggering of the light stimulus.** The signal was band-pass filtered (Texas Instruments FilterPro: center frequency 150 Hz, four poles, *Q* = 1) and the polarity of the filtered signal reversed. When this processed signal exceeded the threshold value defined by the user the device produced a 200-ms TTL pulse and a simultaneous flash of light from the LEDs. The computer and software (E-Prime) used to control the presentation of the conditioning stimuli was also used to block light presentation immediately before and during the training trial via delivering a TTL pulse to this custom-made device.

To detect SPW-Rs online, the signal from the hippocampus was conveyed to a custom-made device where it was band-pass filtered (center frequency 150 Hz, see Figure A1). Next, a simple amplitude threshold for the filtered signal was set. The threshold value varied depending on signal quality, which further depended on the exact location of the electrode tip and the electrode properties. When the amplitude of the band-pass filtered hippocampal signal exceeded the pre-determined threshold, the device triggered a 200-ms pulse of light from three LEDs placed in front of the rabbit. A safety period was set so that SPW-Rs never triggered a light pulse during the training trial, but only during the ITIs. The light stimulus consistently evoked a theta-band response in the hippocampus (see Figures 3 and 5), but the SPW-R itself was left intact and the disruption of oscillatory activity occurred immediately following the SPW-R (see Figure 5).

#### Offline analysis of SPW-Rs

To examine the rate at which hippocampal SPW-Rs were followed by a light stimulus, the LFP signal was analyzed offline. The hippocampal LFP signal from the first conditioning session was first band-pass filtered between 100 and 250 Hz. Next a negative threshold of M – 7 × SD was set and all events exceeding the threshold examined. The ratio (%) between SPW-Rs that were followed by the light stimulus and all SPW-Rs detected offline was calculated. On average 89% of SPW-Rs were detected by our on-line monitoring device. Also, the number in general and timing of SPW-Rs relative to trial offset and previous SPW-Rs in both the R and the Y group were analyzed. Results are presented in Figures 5D–F.

#### Theta-band phase locking

**Figure 2:**
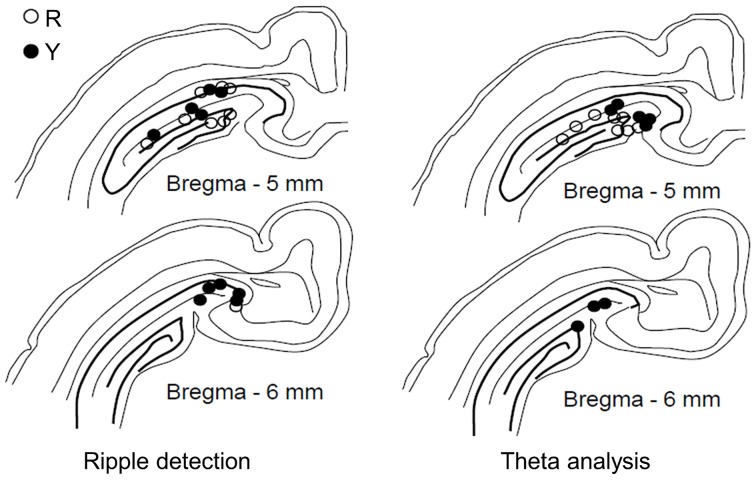
**Placement of recording electrodes in the dorsal hippocampus.** Electrodes used in the yoked control (Y) and SPW-R-contingent (R) groups for detecting ripples (**left**, R: *n* = 9, Y: *n* = 10) and analyzing theta (**right**, R: *n* = 8, Y: *n* = 8). Only animals that learned are included.

Only animals with recording electrodes in the dentate gyrus/hippocampal fissure were included in the analyses on theta activity (see Figure 2). To assess the temporal accuracy of the theta-band response to the conditioning stimuli, a phase-locking value (PLV, see Palva et al., 2005) was calculated. The PLV varies between 0 and 1, 0 indicating no phase locking and 1 indicating perfect phase locking. A simulated data of 60 random samples (× 100,000) of noise generated by a computer (LabView software) was used to estimate the 99th percentile of the PLV distribution in order to evaluate the statistical significance of the real PLVs. Note that the PLV does not depend on the frequency or the amplitude of the signal *per se*, but only on the variability of the phase of the signal from one sweep to another. In the simulated dataset, the 99th percentile was set at PLV = 0.28. The mean value of the PLV was derived from a 500-ms period following the onset of the CS (CS-evoked PLV), the US (US-evoked PLV) or the LED flash (light-evoked PLV) by 200 ms, thus ensuring that the event-related potential was excluded from the analysis.

#### Statistical analyses

Repeated measures analysis of variance (ANOVA) was used in analyzing changes in CR probability with group as a between subjects factor and training session as a within-subjects factor. Similar analyses were run on neural measures (PLV, induced theta, etc.). Whenever a significant interaction of block and group was evident, separate repeated-measures ANOVAs were conducted for each group. Independent samples *t*-test was used in further comparisons between the groups.

### Histology

After the experiments, the rabbits were anesthetized with an i.m. injection of ketamine-xylazine cocktail (see Surgery for details) and then overdosed with an i.v. injection of pentobarbital. Next, the brain was perfused with physiological saline followed by 10% formalin through the ascending aorta. The locations of the electrode tips were marked by passing a DC current (200 μA, 20 s) through the electrode. The brain was then removed and stored in 10% formalin + 30% sucrose solution for approximately 1 week. Next, the brain was frozen and coronally sectioned with a microtome into 100-μm-thick slices. The slices were attached to gelatinized slides and stained with Prussian blue and cresyl violet. The electrode-tip locations were determined from the stained slides with the help of a light microscope and stereotaxic atlas (Bures et al., 1967).

## Results

Animals were implanted with recording electrodes in the dorsal hippocampus (Figure 2) and trained in trace eyeblink conditioning. Each training trial consisted of a 200-ms CS followed by a 100-ms US starting 500 ms (trace period) after the CS-offset. Sixty trials were presented daily for 8 days. Animals in the yoked control (Y) and SPW-R-contingent (R) groups received a light stimulus during the ITI (20–40 s) every time a SPW-R was detected in an animal belonging to the R group, whereas the animals in the normal control group (N) did not receive any light stimuli.

### Hippocampal theta-band responses to the light were similar regardless of preceding neural state

**Figure 3:**
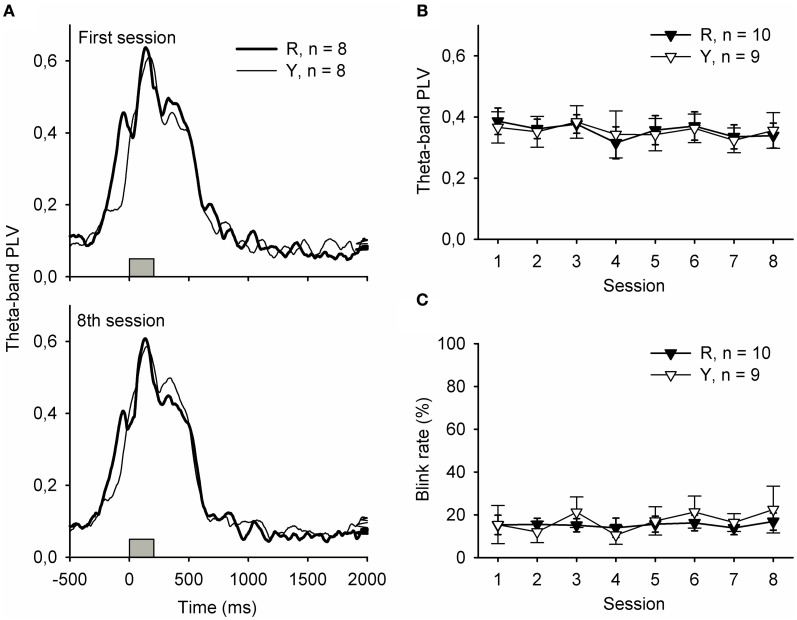
**Hippocampal theta-band responses (A and B) and behavioral responses (C) to the light stimulus were similar in the yoked control (Y) and SPW-R-contingent (R) groups.** In addition, no changes across training were evident. In subplot **(A)**, gray bars indicate the light.

Figure 3 depicts responses to the light stimulus presented during the ITI. Phase-locked hippocampal theta-band responses to the flash of light presented either in contingency with a SPW-R (group R) or irrespective of the ongoing neural state (group Y) were similar, and no changes occurred across training [panels **A** and **B**, repeated measures ANOVA: interaction of group and session: *F*_(7, 149)_ = 0.17, ns., main effect of session: *F*_(7, 149)_ = 0.93, ns., main effect of group: *F*_(1, 17)_ = 0.00, ns.]. Flashing the light during the ITI evoked similar rates of eyeblinks in both the R and the Y groups [panel **C**, repeated measures ANOVA: interaction of group and session: *F*_(7, 149)_ = 1.36, ns., main effect of session: *F*_(7, 149)_ = 0.47, ns., main effect of group: *F*_(1, 17)_ = 0.15, ns.].

### Learning was most disrupted when the light was presented following SPW-Rs

**Figure 4:**
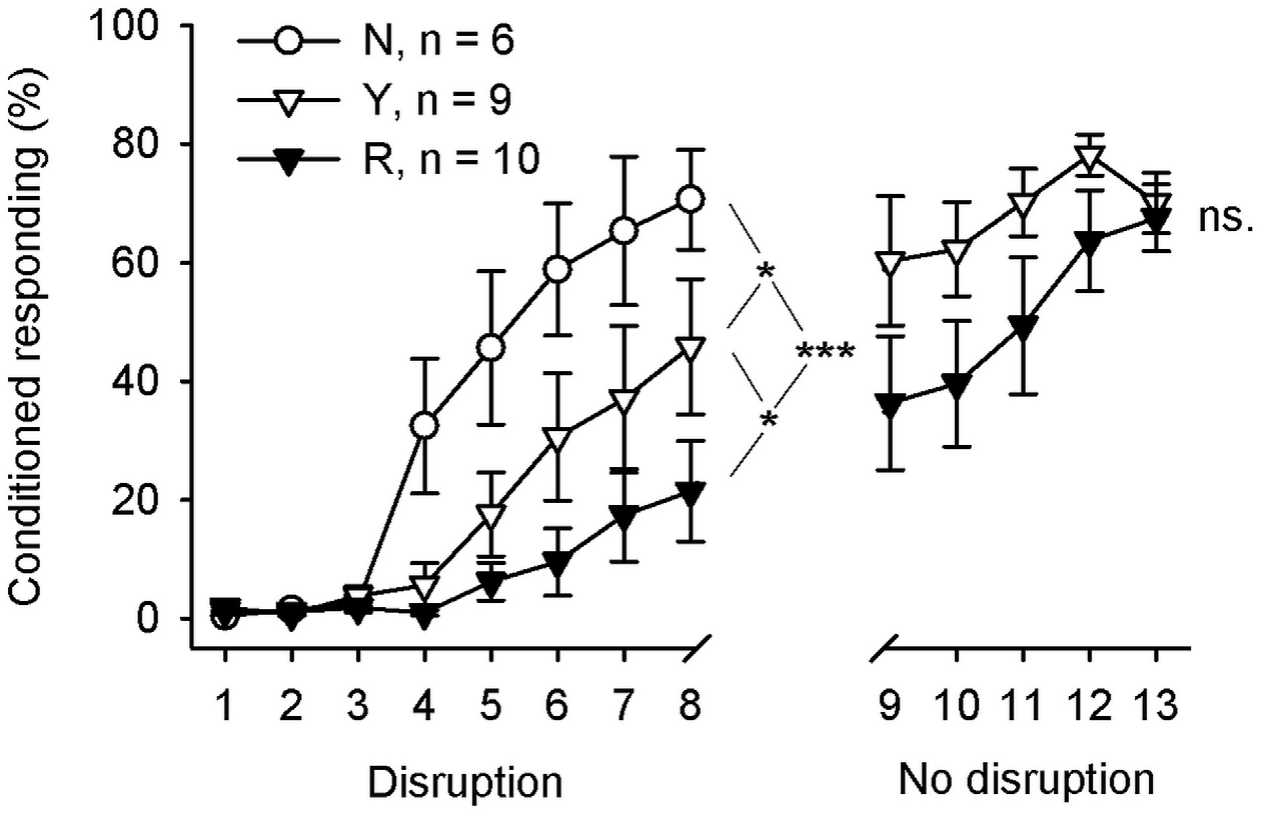
**Flashing a light during inter-trial intervals retarded learning most when done contingent upon hippocampal SPW-Rs.** During the first 8 training sessions, animals in the yoked control (Y) and SPW-R-contingent (R) groups received a light stimulus during the inter-trial interval every time a SPW-R was detected in an animal belonging to the R group (Disruption), whereas the animals in the normal control group (N) did not receive any light stimuli. After that, animals in the yoked control and SPW-R-contingent groups were trained for another 5 days, this time omitting the light (No disruption). Vertical lines depict s.e.m. Asterisks refer to repeated-measures ANOVA group effects: ^*^p < 0.05, ^***^p < 0.001.

The behavioral results are summarized in Figure 4. Learning rate during the initial 8 days of training was analyzed in 3 (groups) × 8 (sessions) repeated measures ANOVA with CR percentage per day as a dependent variable. Learning was significantly different in the 3 groups during the first 8 sessions of training [interaction of session and group: *F*_(14, 154)_ = 4.24, *p* = 0.006; main effect of session: *F*_(7, 154)_ = 31.50, *p* < 0.001; main effect of group: *F*_(2, 22)_ = 7.98, *p* = 0.002]. Subsequent pairwise comparisons between groups revealed that animals trained without the light stimuli (group N, *n* = 6) learned faster than the animals in the SPW-R-contingent group (R, *n* = 10) [*F*_(7, 98)_ = 9.61, *p* < 0.001] or yoked control group (Y, *n* = 9) [*F*_(7, 91)_ = 2.33, *p* = 0.031]. Most importantly, the yoked control group (Y) learned faster than the SPW-R-contingent (R) group [*F*_(7, 119)_ = 2.15, *p* = 0.043]. That is, the light disrupted learning most when presented following ripples.

Immediately, after completion of the first 8 days of conditioning, the animals in the yoked control and SPW-R-contingent groups were trained for another 5 days, this time omitting the light. The animals in both the SPW-R-contingent and yoked control groups now learned equally well [interaction: *F*_(4, 68)_ = 1.05, ns., main effect of group: *F*_(1, 17)_ = 2.58, ns., main effect of session: *F*_(4, 68)_ = 8.04, *p* < 0.001].

### The light did not disrupt the SPW-R but elicited a theta-band response

**Figure 5:**
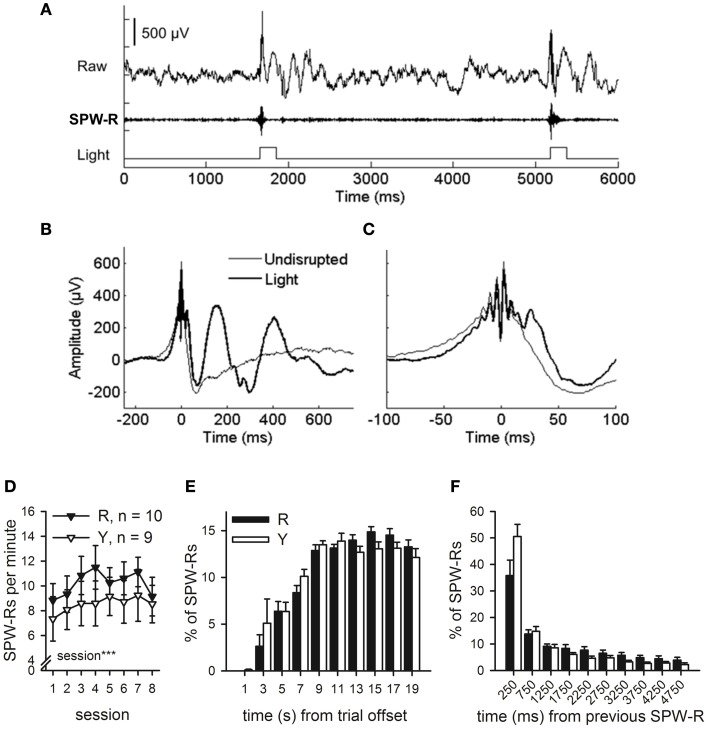
**SPW-R-contingent triggering of light presentations.** Online SPW-R-contingent triggering of light presentations in a representative animal is depicted in **(A)**. In **(B)** and **(C)**, traces represent averages of SPW-Rs recorded during one session in a representative animal (same as in panel **A**). SPW-Rs were either followed by a light (Light) or not (Undisrupted). In **(C)**, a subset of panel **(B)** is presented focusing on the SPW-R. Light did not affect the SPW-R itself, but only the neural activity following it (**B** and **C**). SPW-Rs occurred at a rate of approximately 7–11 per minute **(D)**. More SPW-Rs were observed in both groups as conditioning proceeded (rm ANOVA, main effect of session, *p* < 0.001). Most SPW-Rs occurred 8 or more seconds after the training trial had ceased **(E)**. SPW-Rs tended to occur in close temporal proximity to each other **(F)**. No statistically significant differences between the R and the Y group were observed in the timing of SPW-Rs in relation to training trials or each other. Vertical lines in **(D–F)** depict s.e.m.

Offline analyses of the SPW-Rs indicated that the light presented to the animals in the SPW-R-contingent (*n* = 10) and yoked control (*n* = 9) groups did not disrupt the ripples in the SPW-R-contingent group but rather perturbed the state during and following it (Figures 5B,C). In fact, the number of ripples was comparable in both groups and increased across training [rm ANOVA, interaction: *F*_(7, 119)_ = 1.06, ns., main effect of session: *F*_(7, 119)_ = 4.44, *p* < 0.001, main effect of group: *F*_(1, 17)_ = 0.61, ns.] (Figure 5D). In addition, the distribution of ripples across the ITI and their timing relative to previous ripples was also similar in both the SPW-R-contingent and yoked control groups (Figures 5E,F).

### SPW-R-contingent light presentations disrupted phase-locking of hippocampal theta-band responses to the conditioning stimuli

**Figure 6:**
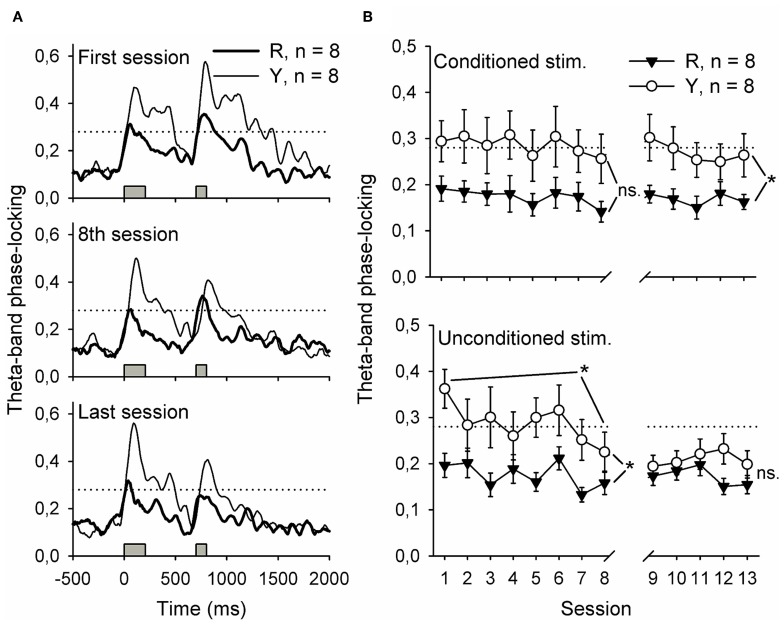
**Flashing a light during inter-trial intervals contingent upon awake-state SPW-Rs disrupted the phase-locking of hippocampal theta-band (4–12 Hz) responses to the conditioning stimuli.** Phase-locking at the beginning of training, at the end of the first phase of training and at the end of all training is depicted in **(A)**. Traces represent grand average of each group. In **(B)**, phase-locking to both stimuli is presented as a function of training session. Note that the values presented in **(B)** are derived from a 500-ms period following the onset of the stimulus by 200 ms, thus ensuring that the event-related potential was excluded from analysis. Gray bars indicate the timing of the conditioning stimuli. Horizontal dotted line indicates the statistical significance (*p* = 0.01) of phase-locking. Vertical lines depict s.e.m. ^*^p < 0.05.

Synchronized oscillatory neural activity might contribute to learning by facilitating efficient signaling between the hippocampus and other brain structures (Sirota et al., 2003, 2008; Wikgren et al., 2010). Indeed, hippocampal theta-band responses to conditioning stimuli reflect the onset of CR acquisition in trace eyeblink conditioning (Nokia et al., 2010). Here, phase-locking of theta-band responses to the CS during the initial eight conditioning sessions was similar in both the yoked control (*n* = 8) and SPW-R-contingent (*n* = 8) groups [Figure 6B top panel, left, rm ANOVA: interaction: *F*_(7, 98)_ = 0.10, ns.; session: *F*_(7, 98)_ = 1.19, ns.; group: *F*_(1, 14)_ = 4.31, ns.]. Phase-locking of responses to the US (Figure 6B lower panel, left) was significantly greater in the yoked control compared to SPW-R-contingent group [group: *F*_(1, 14)_ = 5.40, *p* = 0.036] and decreased in both groups as a function of training [session: *F*_(7, 98)_ = 3.62, *p* = 0.002; interaction: *F*_(7, 98)_ = 1.29, ns.]. When the disruptive light was omitted, the CS evoked a more accurately time-locked theta-band response in the yoked control group compared to the SPW-R-contingent group (see Figure 6 top right panel) [interaction: *F*_(4, 56)_ = 0.37, ns.; session: *F*_(4, 56)_ = 0.77, ns.; group: *F*_(1, 14)_ = 6.56, *p* = 0.023.]. However, the difference in the phase-locking of the theta-band response evoked by the US disappeared (see Figure 6 lower right panel) [interaction: *F*_(4, 56)_ = 0.92, ns.; session: *F*_(4, 56)_ = 0.78, ns.; group: *F*_(1, 14)_ = 2.14, ns.].

To summarize, flashing a light contingent upon hippocampal SPW-Rs led to poorly time-locked theta-band responses to the CS during subsequent training. Theta-band responses to the US paralleled the acquisition of the CR in both groups.

## Discussion

Our results indicate that disrupting large-scale spontaneous brain activity during and immediately following hippocampal SPW-Rs during awake rest periods interspersed with training events interferes with hippocampal processing of those events as they occur and hinders learning overall. Thus, it seems that processing of recently acquired information relying on multiple brain structures such as the hippocampus and the neocortex (see for example Takehara et al., 2003) takes place also outside the immediate SPW-R, although in close contingency with it.

Compatible with the notion that awake-state SPW-Rs are crucial for consolidation (Karlsson and Frank, 2009; Jadhav et al., 2012), disrupting the animal with an external stimulus immediately following SPW-Rs impaired non-spatial, associative, hippocampus-dependent learning. A similar deficit in learning, but to a lesser degree, followed when disruptive stimuli were presented irrespective of the animal's neural state. As the ripple, i.e., fast, high-amplitude oscillation, *per se* was left intact by the presentation of the light, it seems that post-processing of recent events crucial for consolidation takes place not only during hippocampal ripples but also in their absence, during rest periods interspersed with training trials. Although the hippocampal ripples themselves were not affected by the presentation of the light, the communication between the hippocampus and the neocortex (see also Sirota et al., 2003) was probably disrupted, and could explain the deficit in learning.

**Figure A2:**
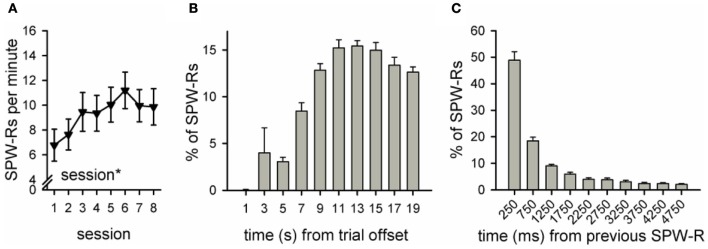
**Number and distribution of ripples in a group of rabbits trained without the light stimulus (*n* = 12, see Nokia et al., 2010).** Data from our previous study (Nokia et al., 2010) yoked control group, first 8 sessions of conditioning was reanalyzed. As in the SPW-R-contingent and yoked control groups in our current study, the frequency of ripples during the inter-trial interval increased across sessions (rm ANOVA, main effect of session *p* < 0.05) and was around 7–11 per minute **(A)**. Also similar to the yoked control and SPW-R-contingent groups in the current study, most SPW-Rs occurred 8 or more seconds after the training trial had ceased **(B)** and SPW-Rs tended to occur in close temporal proximity to each other **(C)**. Note that formatting of this figure (including y-axis scale) is identical to Figure 5 panels **(D–F)**.

Another possible explanation for our results is that the ripple-contingent light presentations disrupted the normal temporal distribution of SPW-Rs in the awake state. During sleep, ripples occur in close contingency with neocortical slow oscillations (Sirota et al., 2003; Molle and Born, 2011). Whether the same is true during awake state, is not known. In any case, in our current study, the distribution of SPW-Rs was virtually the same in the Y and R groups. Also, there was no difference in the total number of SPW-Rs between the Y and R groups. Moreover, both the number and temporal distribution of ripples in both the Y and R groups were highly similar to those of a non-disrupted yoked control group (*n* = 12) from our previous study (see Figure A2 and Nokia et al., 2010). Thus, it seems that the temporal distribution of SPW-Rs was left intact by our experimental manipulation.

Yet another possibility is that in the R group, the animals experienced the light as more distractive because it was presented at a moment when the hippocampus is relatively free from subcortical inhibition (Buzsaki, 1986). This would result in poorer learning, as the animal would pay relatively more attention to the “wrong” stimulus. In fact, we have previously demonstrated that rabbits learn trace eyeblink conditioning faster when the training trials follow ripples, probably because the stimuli used in conditioning elicit a more accurately phase-locked theta-band response in the hippocampus (Nokia et al., 2010). In contrast, the light stimulus we used in this study evoked similar responses whether it was presented immediately following a ripple or not (see Figure 3). Although this might be due to a ceiling effect caused by the higher salience of the light, we still find it improbable that a difference in experiencing the light would be the reason for the difference in learning between the R and Y groups.

Our present results indicate that when left alone to take its natural course, the SPW-R is not followed by any hippocampal oscillatory phenomena that would be consistently time-locked to the SPW-R peak (see Figure 5B). Instead, the presentation of the light (or probably any other external stimulus of sufficient saliency) forces an externally driven oscillatory response in the brain, as evident in the theta-band in the hippocampus. This emphasizes the fact that externally evoked hippocampal theta-band oscillations *per se* are not beneficial for learning (see also Lipponen et al., 2012). Our findings are also in line with the view that endogenous, rest-state brain activity might be essential for consolidation (Buzsaki, 1989). It is clear, that while the discovery of SPW-Rs and their close link to learning is of high importance, we should not stop looking for other mechanisms of memory formation within and beyond the hippocampo-cortical circuit.

In the present study, external stimulation contingent upon hippocampal SPW-Rs changed the immediate hippocampal responses to the stimuli used in conditioning (tone-CS and airpuff-US). Notably, the temporal consistency of the theta-band responses was disturbed, indicating that the hippocampal processing of the stimuli varied from trial to trial instead of reliably following a fixed temporal pattern. It has been suggested that, via synchronized oscillatory activity, it is possible for anatomically distant but functionally related brain structures to communicate effectively (Sirota et al., 2003, 2008). Thus, stimulus-evoked synchronized oscillations at the theta-band, such as those observed here in the yoked control group, might contribute to active encoding phase of learning by repeatedly and predictably forming windows of opportunity for efficient signaling between the hippocampus and other brain structures, for example the neocortex (Sirota et al., 2003, 2008) and the cerebellum (Wikgren et al., 2010). In psychological terms, this would probably translate as attention toward the stimuli.

Most of the knowledge on the learning-related activity patterns of hippocampal cells has been gained by training rats in spatial tasks. Place cells, cells that fire selectively when the animal is in a certain spatial location, are often used as an example of how the hippocampus encodes relational information (see, for example, Foster and Wilson, 2006). However, it has been proposed that the hippocampus is involved in all types of learning that relies on forming associations between discontiguous events or entities (Wallenstein et al., 1998). Indeed, during trace eyeblink conditioning—a non-spatial associative task—a large number of pyramidal cells initially fire as a response to the US, but, at the outset of CR acquisition, start to fire in response to the CS instead (McEchron and Disterhoft, 1997). In addition, some hippocampal cells selectively fire at certain temporal intervals between events relevant for learning (MacDonald et al., 2011). These “time cells” are suggested to bridge the gap between related events in temporally discontiguous tasks. It might be that sequences of time cell firing are also repeated during SPW-Rs.

To summarize, our results together with previous knowledge on the role of SPW-Rs in consolidation suggest that, during multiple-trial learning, the replay and transfer of memory traces from the hippocampus to cortical structures alternates with encoding, and that all of these together are crucial for learning. In addition, it seems that these processes, especially the transfer of information between the hippocampus and cortical structures, depend on neuronal activity related to, but by no means limited to, hippocampal SPW-Rs.

## Conflict of interest statement

The authors declare that the research was conducted in the absence of any commercial or financial relationships that could be construed as a potential conflict of interest.
